# Significant gaps in practice present despite higher levels of public awareness in antibiotic use and antimicrobial resistance in the western province of Sri Lanka

**DOI:** 10.1099/acmi.0.000945.v5

**Published:** 2026-05-05

**Authors:** Conganige Hiranya Hansali Anthony, Anne Sithuvili Hettiarachchige Done, Hettige Kaveesha Sandani Dinudya, Dinushi Tennakoon, Lakshika Lagoshan, Hiripitiyage Gayan Danushka Gunatilake

**Affiliations:** 1 International Institute of Health Sciences (IIHS), Colombo, Sri Lanka

**Keywords:** antibiotic resistance, antimicrobial resistance (AMR), awareness, knowledge, Sri Lanka

## Abstract

**Background.** Antibiotic misuse, influenced by urbanization and limited healthcare access, has accelerated antibiotic resistance, impacting global health. Surveillance in Sri Lanka’s National Strategic Plan for Combating Antimicrobial Resistance (2017–2022) shows significant multidrug resistance in hospitals, with 12.7% of the health budget (3.3 billion) spent on antimicrobials. This resistance complicates treatment and increases patient management costs, particularly in the Western Province, a focal area for analysing antibiotic misuse.

**Objectives.** To assess public awareness of antibiotic misuse and antimicrobial resistance in the Western Province of Sri Lanka.

**Methodology.** A cross-sectional study was conducted on 200 adults in the Western Province from 23 December 2023 to 16 January 2024, using in-person interviews and a Google form questionnaire. Descriptive statistics were applied to analyse the data, and a binary logistic regression analysis was conducted to identify predictors of antimicrobial resistance (AMR) knowledge among participants. The model included sociodemographic, behavioural and awareness-related variables, with statistical significance set at *P*<0.05.

**Results.** Of the respondents, 83% knew antibiotics combat bacterial infections, while 71.5% adhered to completing antibiotic courses. However, 28.5% opposed doing so, with 65.5% stopping antibiotics once they felt better. Regarding antimicrobial resistance, 60.5% recognized the term, and 61% acknowledged that unnecessary antibiotic use could increase bacterial resistance. Participants from the Kalutara District had nearly nine times more good AMR knowledge (AOR=9.10, *P*=0.005), while those earning LKR 20,000–75,000 had almost 11 times more good knowledge (AOR=11.10, *P*=0.033) and those who followed the advice of a health professional had even more knowledge (AOR=851.08, *P*=0.016). Poor knowledge was observed from the rural population (AOR=0.15, *P*=0.006) and those who had ever been infected (AOR=0.21, *P*=0.023). Good knowledge was also present among those who had been divorced or widowed (AOR=12.21, *P*=0.029), had used antibiotics privately and without prescriptions (AOR=4.67, *P*=0.027) or who had heard of AMR but could not remember the source (AOR=51.60, *P*<0.001).

**Conclusion.** Most participants understood antibiotics' role, though gaps in correct use and awareness of misuse consequences persisted. While there was a positive attitude towards antimicrobial resistance, further educational efforts are essential to address knowledge gaps, as recent studies show high resistance levels and limited progress in awareness.

**Impact Statement**

Antimicrobial resistance (AMR) is a growing public health concern in Sri Lanka, driven by limited public awareness and gaps in medication regulation. National surveillance data (2024) indicate elevated resistance rates of E. coli to Cefotaxime (57%) and Meropenem (10.6%), compared to substantially lower rates in the UK. This study assessed public knowledge and practices regarding antibiotic use in the Western Province of Sri Lanka. Although 71.5% (n = 143) of participants reported obtaining antibiotics through appropriate healthcare channels and completing prescribed courses, 65.5% (n = 131) admitted to discontinuing treatment once symptoms improved. These findings demonstrate a disconnect between knowledge and practice, underscoring the need for targeted interventions to promote appropriate antibiotic use and mitigate the progression of AMR.

## Data Summary

The authors confirm that all supporting data, code and protocols have been provided within the article or through supplementary data files. No data were re-used from previous studies.

## Introduction

Antibiotics, upheld as one of the best medical innovations of the twentieth century, have played a paramount role in treating bacteria-based diseases and decreasing mortality rates worldwide. Nonetheless, the misuse of these medications has presented a frightening global health concern – ‘antibiotic resistance’ [1,2]. The development of resistant bacterial forms put in jeopardy the decades of advancement in the practice of medicine, making formerly treatable illnesses potentially fatal. Admitting to the urgency of this problem, public health projects worldwide have strengthened efforts to recognize and address the complicated issue of antibiotic misuse. These efforts are complemented by legislative measures aimed at promoting the safe and rational use of medicines, including antibiotics. For instance, the Sri Lankan National Medicines Regulatory Authority Act No. 5 of 2015 emphasizes the need for responsible medicine distribution and use by healthcare professionals and consumers alike [3]. Additionally, the Sri Lanka National Medicinal Drug Policy outlines key objectives, such as ensuring the availability and affordability of safe and effective medicines, promoting their rational use and encouraging local production of essential medicines. However, despite these regulations, there remains a challenge in the enforcement of these guidelines, as the widespread prescribing and uncontrolled issuing of antibiotics occurs to date at large, leading to ongoing issues with antibiotic misuse. Therefore, strengthening both legislative frameworks and enforcement mechanisms is crucial for effectively mitigating this problem.

From the perspective of this global concern, the Western Province of Sri Lanka serves as a small-scale version where the intricacies of urbanization and healthcare access come together, affecting the pattern of antibiotic usage. With its active urban clusters, rural communities and various socio-economic areas, the Western Province offers an enthralling environment for an intensive analysis into awareness and knowledge of antibiotic misuse in the general population. Furthermore, in relation to the international context, the Western Province represents a relevant study area, being the most populous and economically active region of the country. The coexistence of both advanced healthcare institutions and informal healthcare providers provides complex patterns of antibiotic use and misuse. Being the most populated and economically active area of the nation, it has healthcare characteristics similar to those of many other low- and middle-income countries, such as limited antimicrobial stewardship, inconsistent prescribing practices and easy access to over-the-counter antibiotics [4]. This alignment became apparent as we began our review of existing literature, primarily within Sri Lanka, where we saw a research gap in public awareness studies, most of which fixated on medical professionals and students. To frame our findings in a broader context, we extended our review to include studies from other Asian and South Asian countries with comparable cultural and socioeconomic dynamics. We then associated our findings with those from higher-income countries, where sturdier regulatory frameworks and public health infrastructure frequently influence antibiotic usage patterns differently. In addition to offering important insights into the local causes of resistance, studying antimicrobial resistance (AMR) in this context produces evidence that can be applied to comparable urbanized, resource-constrained environments worldwide, supporting larger initiatives to comprehend and mitigate AMR globally.

Antibiotic resistance, a major component of AMR, occurs when bacteria evolve mechanisms that permit them to resist the effects of antibiotics, making these medicines less effective or even ineffective. AMR covers resistance among bacteria, viruses, fungi and parasites to antibiotics, antivirals, antifungals and antiparasitic drugs, challenging the ability to treat infections effectively. In the Western Province of Sri Lanka, the aspects of antibiotic use are shaped by a variety of factors. The urban landscape, better healthcare accessibility and a mix of modern and traditional healthcare methods create an intricate scene that influences how antibiotics are acquired, prescribed and used. Comprehending the effects of these dynamics is essential for shaping interventions that echo the unique needs of the local populace.

The misuse of antibiotics frequently stems from an absence of awareness about their appropriate use and probable consequences [5,7]. As the whole world struggles with the mounting threat of AMR, this study in the Western Province of Sri Lanka worked towards contributing to localized understandings that may support necessitated changes. To the best of our knowledge, the available literature on awareness regarding antibiotic use and antimicrobial resistance in Sri Lanka predominantly focuses on healthcare professionals. As this emphasis introduces a significant bias, potentially leading to an overestimation of general awareness, this study seeks to explore public awareness more comprehensively. However, while there is some level of awareness about antibiotics and antimicrobial resistance within the general population, preliminary findings indicate that there remains a significant gap in understanding their correct usage and the full implications of misuse.

Although antimicrobial resistance is a more general term that includes resistance in bacteria, viruses, fungi and parasites to a variety of antimicrobial agents, antibiotic resistance particularly refers to the capacity of bacteria to resist the effects of antibiotics. While our overarching objective of addressing the problem of AMR, pragmatic factors, such as accessibility and resource limitations, have led us to concentrate mostly on examining antibiotic misuse and how it contributes to the emergence and progression of AMR.

### General objective

The overarching goal of this research is to assess the level of public knowledge and awareness of antibiotic use and antimicrobial resistance in the Western Province of Sri Lanka.

### Specific objectives

Determine the level of public knowledge on antibiotic use in the Western Province of Sri Lanka.

Evaluate the level of public knowledge on antimicrobial resistance in the Western Province of Sri Lanka.

Investigate the factors influencing antibiotic use among the population in the Western Province.

Explore the awareness levels of the consequences of antimicrobial resistance in the Western Province.

Assess the overall level of public knowledge on both antibiotic use and antimicrobial resistance in the Western Province.

## Methods

### Study design

A cross-sectional descriptive study method was carried out.

#### Study setting

This study was conducted among the population of the western province of Sri Lanka. The population was within the age range of 18 years old to 70 years old. Data was collected from 23 December 2023 to 16 January 2024 with the use of an interviewer-administered questionnaire. Participants were approached in public locations across the three districts of Colombo, Gampaha and Kalutara.

Data collectors aimed to recruit participants from each district to ensure representation from all three administrative areas of the Western Province. Although formal quotas were not imposed, efforts were made to approach individuals from diverse age groups, genders and residential settings (urban and rural) to enhance variability within the convenience sample.

#### Study population

The study population was defined as adults (aged 18–70 years), residing in the districts of Colombo, Gampaha and Kalutara in the Western Province of Sri Lanka. Participants were not limited by educational background or prior experience with antibiotics.

### Inclusion criteria

- Individuals who live in the Western Province of Sri Lanka.
- Participants aged 18 to 70 years old.
- Individuals who consented to participate in the study.

### Exclusion criteria

- Individuals who live outside the Western Province of Sri Lanka.
- Participants younger than 18 years old and older than 70 years old.
- Individuals who did not consent to participate in the study.

#### Study instrument

An interviewer-administered questionnaire was established following an extensive review of published literature. The tool was adapted from a study conducted in Ethiopia titled ‘Assessment of Public Awareness, Attitude and Practice Regarding Antibiotic Resistance in Kemissie Town, Northeast Ethiopia: Community-Based Cross-Sectional Study’ [8]. This survey was selected as the baseline as it was easily adaptable to our study context and, therefore, convenient to modify for use in Sri Lanka.

The questionnaire collected demographic information, including age, gender, marital status, occupation, residential area, level of education and income level, as well as data related to antibiotic use and antimicrobial resistance. By adapting this previously tested tool, we ensured methodological validity while modifying it to the healthcare and sociocultural dynamics of Sri Lanka’s Western Province. The questionnaire was prepared in the three primary languages spoken in Sri Lanka, English, Sinhala and Tamil, and participants were allowed to choose their preferred language. Interviews were led in the language preferred by the individual participants to improve comprehension and guarantee comfort during the process. Responses were entered directly by the interviewer into a Google Form linked to a central Google Sheet to help with the easy consolidation of results from all interviewers. This allowed for reliable data entry and well-organized digital consolidation.

To ensure local relevance, a pre-test of the questionnaire was conducted within the Western Province using a small group of university students, as the research was carried out as part of an undergraduate course module. Participants were selected through convenience sampling in the areas of Colombo, Gampaha and Kalutara and completed the questionnaire in the same manner as in the main study, through interviewer administration. Verbal and written feedback were collected regarding question clarity, length and language comprehension. Based on this feedback, minor revisions were made to simplify wording, remove redundant items and ensure culturally appropriate phrasing. This pre-test helped improve question wording and response options, verify cultural appropriateness and improve the tool’s validity and applicability for the main study.

### Data analysis

Descriptive statistics (frequencies and percentages) summarized participants’ sociodemographic characteristics and responses related to AMR. Antimicrobial resistance knowledge was assessed using 30 structured items related to awareness, causes and consequences. Each correct response was awarded one point, while incorrect or ‘don’t know’ responses were scored as zero. The total knowledge score ranged from 0 to 30. Participants scoring ≥50% of the total possible score were categorized as having ‘good knowledge’, while those scoring below 50% were categorized as having ‘poor knowledge’. This cutoff was selected based on commonly applied thresholds in similar cross-sectional knowledge studies. Chi-square (*χ*²) tests examined associations between key sociodemographic variables and AMR awareness and antibiotic use. Binary logistic regression identified predictors of adequate AMR knowledge, with results reported as adjusted odds ratios (AORs) and 95% CIs. Model adequacy was evaluated using the omnibus *χ*², *R*² values, classification accuracy and the Hosmer–Lemeshow test. A sensitivity analysis, conducted by collapsing sparse categories and refitting the model, confirmed the stability and robustness of the main predictors. Statistical significance was set at *P*<0.05, and all analyses were performed using IBM SPSS Statistics version 26.

## Results

### Socio-demographic characteristics

As per Table 1, out of 200 total respondents, the majority of the participants (52%) were females, 42% were from the Gampaha district, 33% were from the Colombo district and 25% were from the Kalutara district. 33.5% of the respondents were in the age group of 18–25 years, and the vast majority (42%) were from the Gampaha District. The majority of participants (27%) selected their education level as a bachelor’s degree, with 30.5% selecting 20,000–75,000 as the average income.

**Table 1.** Socio-demographic characteristics of respondents in Gampaha District, Sri Lanka, January 2024 (*N*=200)

Socio-demographic characteristic	Category	Frequencies (*n*)	Percentage (%)
What is your gender? (*n*=200)	Male	96	48
	Female	104	52
Age group (*n*=200)	18–25	67	33.5
	26–35	35	17.5
	36–45	39	19.5
	46–55	32	16
	56 and above	27	13.5
District of the Western Province (*n*=200)	Colombo	66	33
	Gampaha	84	42
	Kalutara	50	25
Type of residence (*n*=200)	Urban	122	61
	Rural	78	39
Marital status (*n*=200)	Married	91	45.5
	Unmarried	89	44.5
	Divorced	4	2
	Widowed	7	3.5
	Prefer not to say	9	4.5
Education level (*n*=200)	Less than O/L	17	8.5
	O/L	24	12
	A/L	49	24.5
	Bachelor’s degree	54	27
	Postgraduate degree	29	14.5
	Master’s level	20	10
	PhD	5	2.5
	Prefer not to say	2	1
Occupation (*n*=200)	Manager	11	5.5
	Healthcare professional	20	10
	Technicians and associate professionals	7	3.5
	Engineer/technical professional	21	10.5
	Clerical support workers	10	5
	Self-employed/entrepreneur	12	6
	Service and sales workers	9	4.5
	Skilled agricultural, forestry and fishery workers	7	3.5
	Craft and retail trades workers	8	4
	Plant and machine operators and assemblers	7	3.5
	Other	73	36.5
	Prefer not to say	15	7.5
Average monthly income (*n*=200)	<20,000	28	14
	20,000–75,000	61	30.5
	75,000–200,000	45	22.5
	>200,000	24	12
	Prefer not to say	42	21

### Sources and practice of antibiotics

The vast majority of the respondents (94%) took antibiotics, as mentioned in Table 2. Of these, 71.5% (*n*=143) obtained the antibiotics from hospitals/healthcare by prescription, respondents 20% (*n*=40) from retail outlet pharmacies, whereas 15 (7.5%) obtained the antibiotics from a friend or family member. 83% (*n*=166) of participants acknowledged that they obtained antibiotics with the intent to cure a bacterial infection. The majority of participants, 193 (96.5%), agreed to follow medical professionals’ advice when taking antibiotics, with 188 participants (94%) having verbal or written advice on how the prescribed antibiotic is to be used. However, unfortunately, 131 (65.5) participants confessed to the habit of terminating the use of antibiotics when they start to feel better.

**Table 2.** Distribution of antibiotic usage factors of the study participants

Antibiotic usage factor	Category	Frequencies (*n*)	Percentage (%)
Have you ever used antibiotics? (*n*=200)	Yes	188	94
	No	09	4.5
	I don’t know	02	1
	Don’t remember	01	0.5
Antibiotics can kill bacteria (*n*=200)	Yes	166	83
	No	13	6.5
	I don’t know	16	8
	Don’t remember	05	2.5
How do you typically obtain antibiotics? (*n*=200)	Hospital/healthcare by prescription	143	71.5
	Retail outlet pharmacy	40	20
	From a friend or family member	15	7.5
	By sharing with others	02	1
When prescribed antibiotics, do you generally follow the health professional's advice? (*n*=200)	Agree	193	96.5
	Disagree	07	3.5
Were you provided verbal or written information about how often the antibiotic to take? (*n*=200)	Agree	189	94.5
	Disagree	11	5.5
Were you provided verbal or written information about how much of the antibiotic to take? (*n*=200)	Yes	188	94
	No	12	6
Do you make it a point to finish the antibiotic course as advised by the health professional? (*n*=200)	Agree	143	71.5
	Disagree	57	28.5
Do you consider the recommended time gap between antibiotic doses? (*n*=200)	Agree	149	74.5
	Disagree	51	25.5
If you start feeling better after a few days, do you stop taking antibiotics? (*n*=200)	Agree	131	65.5
	Disagree	69	34.5
Have you ever stopped taking antibiotics due to a deviation from the normal schedule? (*n*=200)	Agree	113	56.5
	Disagree	87	43.5
Have you ever stopped taking antibiotics due to negligence, being fed up or bored? (*n*=200)	Agree	104	52
	Disagree	96	48
Have you ever taken antibiotics without a prescription? (*n*=200)	Yes	97	48.5
	No	103	51.5
Have you experienced different microbial infections during your lifetime? (*n*=200)	Yes	159	79.5
	No	41	20.5
How long does a typical microbial infection last? (*n*=200)	Less than a day	12	6
	2 to 5 days	71	35.5
	1–2 weeks	59	29.5
	More than 2 weeks	25	12.5
	I can’t remember	33	16.5
If you've had microbial infections, did you take antibiotics to treat them? (*n*=200)	Yes	169	84.5
	No	31	15.5

### Awareness of antibiotic resistance

As shown in Table 3, 60.5% (*n*=121) of respondents understood the term ‘antimicrobial resistance’, and 61% agreed that unnecessary antibiotic use increases bacterial resistance. Of these respondents, 48% (*n*=96) learnt about it from healthcare professionals, 25.5% (*n*=51) from mass media, 7.5% (*n*=15) from friends, while 19% (*n*=38) did not remember. Additionally, 76.5% (*n*=153) agreed that antibiotic resistance is a global issue. Contributing factors cited included overuse (53.5%), underuse (43%), incomplete therapy (34%), sharing antibiotics (47.5%), taking without prescription (42%) and improper dosage/timing (42%). Only 8% selected ‘others’.

**Table 3.** Distribution of antibiotic resistance factors of the study population

Antimicrobial resistance factor	Category	Frequencies (*n*)	Percentage (%)
Do you know what the term ‘Antimicrobial resistance’ means (*n*=200)	Yes	121	60.5
	No	79	39.5
How have you heard or encountered the term ‘Antimicrobial resistance’ before? (*n*=200)	Healthcare professional	96	48
	Mass media	51	25.5
	Friends	15	7.5
	Don’t remember	38	19
Do you believe that the development of antimicrobial resistance is a problem? (*n*=200)	Agree	156	78
	Disagree	44	22
Can the unnecessary use of antibiotics increase the resistance of bacteria to them? (*n*=200)	Agree	122	61
	Disagree	78	39
Do you agree that resistance to antibiotics is a worldwide problem? (*n*=200)	Agree	153	76.5
	Disagree	47	23.5
What do you think are the risk factors of antibiotic resistance? (*n*=200)	Over or underuse of antibiotics	107	53.5
	Failure to complete the course of therapy	86	43
	Sharing antibiotics with others	68	34
	Taking antibiotics without a prescription	95	47.5
	Taking antibiotics without considering the dose and time gap	84	42
	Others	16	8
What consequences do you associate with antimicrobial resistance? (*n*=200)	Decrease antibiotic activity	112	56
	Need for expensive drugs	62	31
	Not cured from diseases	79	39.5
	Increases the intensity and duration of diseases	94	47
	Others	29	14.5

### Knowledge of antibiotic resistance factors

With regard to the participants who understood the consequences associated with antimicrobial resistance (*n*=112), 56% participants believed that the decrease of antibiotic resistance was the major consequence, while 62 respondents, equalling 31% of the population, believed that the need for expensive drugs could be a potential consequence of antimicrobial resistance. 39.5% (*n*=79) and 47% (*n*=94) of the respondents believed that it could result from not curing diseases and that it could increase the intensity and duration of diseases, respectively.

**Fig. 1.**
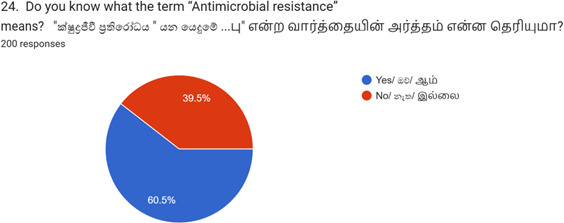
Knowledge of the term ‘antimicrobial resistance’ among respondents (*N*=200).

In general, there was a high level of awareness and knowledge observed. Nonetheless, issues were noted in the practical application of antibiotic use, particularly with individuals discontinuing antibiotics once they started feeling better. Overall, 60.5% of respondents reported awareness of the term ‘antimicrobial resistance’, while 39.5% did not. Additionally, 65.5% of participants reported discontinuing antibiotics once they started feeling better (Fig. 1).

### Associations between demographics and antibiotic use/AMR awareness

Chi-square analysis revealed several statistically significant associations between demographic factors and antibiotic-related knowledge and practices. A significant relationship was found between educational level and awareness of antimicrobial resistance (*χ*²=17.42, *df*=3, *P*=0.001), where respondents with higher education were more likely to be aware of AMR. Similarly, gender was significantly associated with whether participants completed the full course of antibiotics (*χ*²=9.92, *df*=1, *P*=0.002), with females more likely to complete the course. Employment status was significantly related to knowledge that antibiotics are for bacterial infections (*χ*²=6.47, *df*=2, *P*=0.039). No significant associations were observed between age group and most knowledge or attitude variables (*P*>0.05), as summarized in Table 4.

**Table 4.** Associations between demographic variables and key knowledge/Practice Outcomes

Demographic variable	Knowledge/practice variable	*χ*²	*df*	***P*-value**	Significance
Education level	Awareness of antimicrobial resistance	17.42	3	0.001	Yes
Gender	Completion of full antibiotic course	9.92	1	0.002	Yes
Employment status	Knowledge that antibiotics treat bacterial infections	6.47	2	0.039	Yes
Age group	Awareness of antibiotic misuse consequences	2.81	2	0.245	No
Gender	Awareness of AMR	0.74	1	0.389	No

### Predictors of adequate knowledge on antimicrobial resistance

**Fig. 2.**
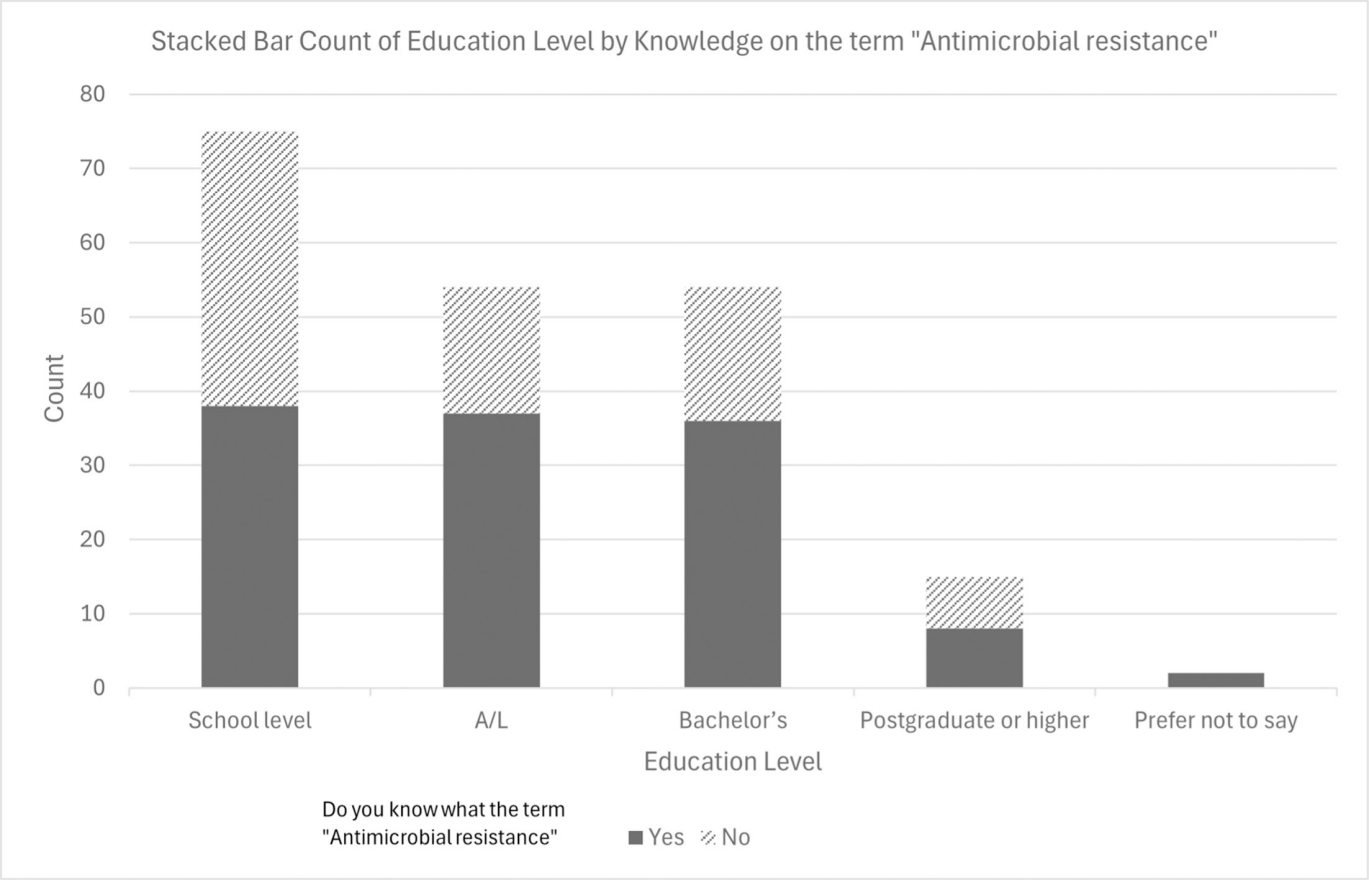
Knowledge of the term ‘antimicrobial resistance’ by education level (*N*=200). A stacked bar chart showing the distribution of respondents’ knowledge of the term ‘antimicrobial resistance’ across different education levels. ‘Yes’ responses (grey filled) and ‘No’ responses (diagonal lines) are stacked by count. The highest proportion of knowledge is observed among respondents with higher education.

A binary logistic regression was performed on the participants’ knowledge on the subject of AMR to determine the predictors of sufficient knowledge on the subject of AMR. The dependent variable was knowledge of AMR, which was binary coded 1=good knowledge, 0=poor knowledge. The independent variables were age group, gender, marital status, education, occupation, income, district, place of residence, history of infection, patterns of behaviour regarding antibiotic consumption and some other awareness-related variables. The model was highly significant, *χ*² (47, *N*=200)=129.49, *P*<0.001, at the same time the model explained 47.7% (Cox and Snell *R*²) to 64.5% (Nagelkerke *R*²) of the variance on AMR knowledge and was able to classify 86.5% of the cases. The model achieved considerable predictive accuracy and the Hosmer–Lemeshow test showed good fit, *χ*² (8)=4.31, *P*=0.828. After adjusting for all covariates, several predictors were significant (Table 5). Participants from Kalutara District had better odds of good knowledge compared to those from Colombo [AOR=9.10, 95% CI (1.94, 42.71), *P*=0.005]. Respondents earning LKR 20,000–75,000 per month had higher odds of adequate knowledge compared to those earning below LKR 20,000 [AOR=11.10, 95% CI (1.21, 101.93), *P*=0.033]. Participants who followed the advice of health professionals when antibiotics were prescribed tended to have better knowledge [AOR=851.08, 95% CI (3.58, 202,244.70), *P*=0.016]. Living in rural areas, in contrast, had lower odds of adequate knowledge compared to those in urban areas [AOR=0.15, 95% CI (0.04, 0.58), *P*=0.006] and those reporting multiple past infections had lower knowledge [AOR=0.21, 95% CI (0.05, 0.80), *P*=0.023]. Divorced, widowed and those who chose not to state their marital status had better odds than unmarried participants [AOR=12.21, 95% CI (1.30, 114.73), *P*=0.029] and so did participants who took antibiotics without medical prescriptions [AOR=4.67, 95% CI (1.20, 18.23), *P*=0.027] and those who have heard of AMR and do not remember the source [AOR=51.60, 95% CI (9.10, 292.60), *P*<0.001]. The model also included gender, age group, education(Fig. 2) level and occupation, and although their contributions were not statistically significant (*P*>0.05), they have greater importance for the stability and interpretability of the model. Sensitivity analysis confirms the stability and robustness of the main predictors by collapsing sparse categorical levels and refitting the model, which showed only minor reductions in extreme odds ratios.

**Table 5.** AOR from multivariable logistic regression predicting adequate AMR knowledge (*N*=200)

Predictor (reference in *italics*)	AOR	95%** CI**	*P*
**Gender (** ***male*** **)**			
Female	0.41	0.13–1.27	0.120
**Age group (*18–25*)**			
26–35	1.77	0.21–15.24	0.604
36–45	2.44	0.23–26.16	0.461
46–55	4.32	0.38–48.86	0.237
56+	3.88	0.37–40.62	0.258
**District (Western Province) (*Colombo***)			
Gampaha	0.90	0.18–4.52	0.902
Kalutara	9.10	1.94–42.71	0.005
**Residence (*urban***)			
Rural	0.15	0.04–0.58	0.006
**Marital status (*unmarried***)			
Married	2.10	0.51–8.65	0.307
Divorced/widowed/prefer not	12.21	1.30–114.73	0.029
**Education level** (***< O/L***)			
O/L	0.88	0.26–3.01	0.840
A/L	1.26	0.22–7.11	0.796
Bachelor’s	0.56	0.06–5.52	0.619
Postgraduate	—	—	0.999
**Occupation (*Mgr/HC/Eng/Tech***)			
Clerical/tech/associate	0.49	0.06–3.77	0.491
Service/sales/self-emp.	0.36	0.04–3.08	0.354
Plant/agri/craft	1.61	0.23–11.13	0.627
Other/prefer not	1.63	0.26–10.14	0.601
**Monthly income** (***<20,000* LKR**)			
20,000–75,000	11.10	1.21–101.93	0.033
75,000–200,000	1.76	0.15–20.24	0.650
>200,000	0.19	0.01–3.63	0.272
Prefer not	1.15	0.15–8.75	0.895
**Ever used antibiotics (*no***)	0.08	0.01–1.06	0.055
**‘Antibiotics can kill bacteria’ (agree**)	3.44	0.71–16.80	0.126
**How antibiotics are obtained (*hospital/by prescription***)			
Retail pharmacy	0.84	0.25–2.83	0.772
Friend/family	5.78	0.53–62.74	0.150
Sharing with others	—	—	0.999
**Follow health-professional advice (when prescribed) (*no/unsure***)	851.08	3.58–202,244.70	0.016
**Info provided: how often (*no***)	0.17	0.01–6.55	0.344
**Info provided: how much (*no***)	0.09	0.00–2.45	0.150
**Finish full course (*no***)	0.84	0.19–3.76	0.820
**Consider time gap (*no***)	0.61	0.15–2.56	0.504
**Stop if feeling better (*no***)	1.23	0.37–4.11	0.742
**Stopped due to schedule deviation (*no***)	2.52	0.78–8.18	0.123
**Stopped due to negligence/boredom (*no***)	1.18	0.32–4.36	0.807
**Taken antibiotics without a prescription (*no***)	4.67	1.20–18.23	0.027
**Experienced different infections (lifetime) (*no***)	0.21	0.05–0.80	0.023
**Typical infection length (*<1* day**)			
2–5 days	0.16	0.02–1.20	0.074
1–2 weeks	0.31	0.04–2.51	0.270
>2 weeks	0.43	0.05–4.16	0.468
Can’t remember	0.38	0.03–4.29	0.432
**Heard/encountered ‘AMR’ (*healthcare professional***)			
Mass media	1.88	0.52–6.90	0.339
Friends	1.80	0.29–11.36	0.532
Don’t remember	51.60	9.10–292.60	<0.001

Bold indicates *P*<0.05. Dashes denote unstable/undefined CIs caused by sparse cells (quasi-separation). Reference categories are italicized. The final binary logistic regression model was statistically significant, *χ*² (47, *N*=200)=129.49, *P*<0.001, explaining 47.7% (Cox and Snell *R*²) to 64.5% (Nagelkerke *R*²) of the variance in AMR knowledge and correctly classifying 86.5% of cases. The Hosmer–Lemeshow goodness-of-fit test was non-significant (*χ*² (8)=4.31, *P*=0.828), confirming good model fit.

## Limitations

### Regional scope

This study was limited to the Western Province of Sri Lanka, a relatively more developed region, which may affect the generalizability of findings to less developed areas with differing healthcare access and education levels.

### Sample size and study context

As the study was conducted due to an undergraduate research module, the sample size was limited to 200 participants due to time and logistical constraints. Regardless of this, the inclusion of participants from all three districts provides a useful baseline for future, larger-scale studies.

Although the questionnaire was pre-tested among university students within the Western Province, this group may not fully represent the broader target population in terms of age distribution, educational background and socioeconomic diversity.

As university students may have higher baseline health literacy, the pilot testing process may not have captured comprehension challenges that could arise in the general population.

### Data collection period

The short data collection period stemmed from academic and ethical approval deadlines as the study was conducted due to an undergraduate research module. While this limited the temporal scope, the study still offers a significant snapshot of public awareness during the period, serving as a reference point for future research.

Due to the nature of convenience sampling and interviewer-administered data collection, the exact number of individuals who declined participation was not systematically recorded. However, incomplete responses were minimal, and only fully completed questionnaires (*n*=200) were included in the final analysis.

### Study design

A cross-sectional descriptive design was used to swiftly assess awareness levels within a defined population. While appropriate for exploratory purposes, it does not portray behavioural trends or changes over time. Future longitudinal studies are recommended to study these dynamics further.

### Sampling method

Convenience sampling was employed due to time constraints, allowing efficient data collection but introducing potential selection bias. Underrepresentation of rural or marginalized populations may have biassed results towards more educated or health-aware participants. Probability-based sampling in future studies could improve representativeness.

For the logistical regression, predictor variables were selected based on theoretical relevance, prior literature and variables demonstrating significance in bivariate analysis (*P*<0.20). Given the relatively small sample size, we acknowledge that including multiple predictors may have reduced model stability and contributed to wide confidence intervals in some estimates. Therefore, results, particularly those with large odds ratios and wide confidence intervals, should be interpreted cautiously.

## Discussion

### Antibiotic usage

This study assessed the public’s knowledge and awareness of antibiotic usage and antimicrobial resistance in the Western Province of Sri Lanka. Among respondents, 71.5% stated that they typically obtain antibiotics from a hospital or healthcare facility, and an equal percentage reported that they complete the full antibiotic course as advised by a medical professional. Yet, a noteworthy contradiction was identified: 65.5% later admitted to discontinuing their antibiotic course once they began to feel better. This inconsistency highlights a disconnect between knowledge and behaviour, where an apparent understanding of antibiotic use does not always lead to correct practice.

One likely explanation is the lack of targeted education on the importance of completing antibiotic courses and the dangers of misuse. While participants may have met with general health messaging, in-depth and constant public education appears inadequate. Behavioural patterns may also be moulded by generational beliefs or misinformation, such as the impression that ‘too much medication is harmful’ or a fondness for a ‘quick fix’ approach. Fascinatingly, high rates of misuse were observed across all income levels. For lower-income individuals, this may be due to barriers in affordability or inadequate access to healthcare, while in higher-income groups, overconfidence in self-medication or a lower awareness of risk may contribute.

When compared to international studies (Table 6), similar discrepancies were observed. In Kemissie Town, Northeast Ethiopia, 74.7% of respondents had obtained antibiotics with a prescription, while only 51.5% of participants in the present study reported doing so. Other studies recorded 73.1% in Ghana, 54.2% in Pakistan and 36.4% in Saudi Arabia, obtaining antibiotics via prescription [9,11]. Remarkably, in the Saudi Arabia study, the capability to purchase antibiotics without a prescription was regarded positively, as it enabled self-medication [10]. In contrast, a study among Sri Lankan pharmacy students found that 77% obtained antibiotics by prescription [12], signifying a possible effect of formal education.

**Table 6.** Comparisons of all studies discussed in the article with the present study By comparing the findings of this study to other countries in South Asia, it was made possible to contextualise the results within a broader international landscape.

Question		Current study, *n* (%)	Chandrakanth *et al*. [17], ***n* (%**)	Mengesha *et al*. [9], *n* (%)	Raihan *et al*. [18], ***n* (%**)	Abdel-Qader *et al*. [14]**, *n* (%**)	Khan *et al*. [12], ***n* (%**)	Effah *et al*. [10]**, *n* (%**)	Shah *et al*. [22], ***n* (%**)	Jayaweerasingham *et al*. [13], ***n* (%**)	Siltrakool *et al*. [16]**, *n* (%**)
Antibiotics can kill bacteria?	Yes	166 (83%)	110 (61.11%)	nd	30–40%	310 (98.4%)	nd	nd	1057 (86.9%)	182 (91.5%)	nd
	No	13 (6.5%)	22 (12.22%)		60–100%	5 (1.6%)			160 (13.1%)	17 (8.5%)	
	I don’t know	16 (8%)	48 (23.33%)		nd	nd			nd	nd	
	Don’t remember	5 (2.5%)	nd		nd	nd			nd	nd	
How do you typically obtain antibiotics?	Hospital/healthcare by prescription	143 (71.5%)	nd	258 (74.8%)	nd	nd	52 (54.2%)	241 (75%)	nd	nd	nd
	Retail outlet pharmacy	40 (20%)		14 (4%)			41 (42.7%)	nd			
	From a friend or family member	15 (7.5%)	56 (31.11%)	nd		310 (6.1%)	3 (3.1%)	186 (58%)			
	By sharing with others	2 (1%)	nd	7 (1.8%)		nd	nd	nd			
If you start feeling better after a few days, do you stop taking antibiotics?	Agree	131 (65.5%)	55 (30.56%)	71 (75.5%)	12%	310 (79.4%)	nd	136 (21.5%)	1039 (85.2%)	64 (72.2%)	nd
	Disagree	69 (34.5%)	125 (69.44%)	23 (24.5%)	88%	80 (20.6%)		307 (78.5%)	181 (14.8%)	25 (27.8%)	
Have you ever taken antibiotics without a prescription?	Yes	97 (48.5%)	138 (76.66%)	122 (35.67%)	51%	310 (99.7%)	23 (24%)	nd	nd	nd	160 (43.0%)
	No	103 (51.5%)	42 (23.33%)	220 (64.33%)	50%	1 (0.3%)	73 (76%)				212 (57%)
Do you know what the term ‘antimicrobial resistance’ means	Yes	121 (60.5%)	nd	171 (77.03%)	90%	nd	26 (27.1%)	nd	901 (73.9%)	nd	nd
	No	79 (39.5%)		51 (22.97%)	10%		70 (72.9%)		318 (26.1%)		
Do you agree that resistance to antibiotics is a worldwide problem	Agree	153 (76.5%)	nd	154 (41.6%)	nd	nd	nd	129 (20.41%)	nd	nd	261 (70.2%)
	Disagree	47 (23.5%)		216 (58.4%)				503 (79.6%)			111 (29.8%)

nd, not discussed.

Concerning adherence to antibiotic courses, 71.5% of our participants stated that they completed their prescribed treatment. Yet, only 34.5% disagreed with stopping antibiotics if symptoms improved after a few days. This mirrors the findings from other populations: 79.4% of participants in a Jordanian study completed the course [13], and over 65% did the same in Thailand [14]. In Sri Lanka, a study among undergraduate nursing students stated that more than 80% understood antibiotics must be taken at correct intervals and for the prescribed duration [15]. Likewise, 85.2% of respondents in Nepal, 88% in Bangladesh and 69.4% in India opposed discontinuing antibiotics early [16,18]. These findings propose that the practice of ceasing antibiotics early is widespread and not always lessened by awareness alone.

Added insights from our study show that while 74.5% of participants considered the suggested time gap between doses, only 43.5% avoided discontinuing antibiotics due to schedule deviation. Moreover, 48% did not stop due to negligence, boredom or frustration. In contrast, the Kemissie Town study recorded 86.4% adherence to dose timing, 22.3% discontinuing due to time deviation and 36.2% due to boredom. These comparisons show that public knowledge about proper antibiotic use remains insufficient in the Western Province.

Taken together, the findings of this study, along with related international research, highlight a worrying pattern of inconsistent adherence to antibiotic guidelines. In spite of varying levels of awareness and healthcare access, a considerable portion of individuals across different regions fails to complete prescribed antibiotic courses, frequently discontinuing prematurely when symptoms improve. This behaviour continues even among those obtaining antibiotics through formal healthcare channels, strengthening the point that knowledge does not automatically translate into responsible practice.

### Antimicrobial resistance

In this study, 60.5% of participants understood the term ‘antimicrobial resistance’. This level of awareness is comparatively low in comparison to other populations. In Ethiopia, for instance, 77% of respondents reported knowing what AMR means. In the UK, awareness was even higher, with 96% strongly agreeing that they understood the concept. In South Asia, awareness varied: 90% of respondents in Bangladesh, 73.9% in Nepal, and only 27.1% in Pakistan reported understanding the term [11,17, 18].

Within Sri Lanka, AMR awareness among healthcare students is pointedly higher than in the general public. A study among university pharmacy students stated that 90% were knowledgeable about AMR, with 78% recognizing it as a serious problem [12]. Likewise, a study on undergraduate nursing students stated that 99.4% believed inappropriate antibiotic use could lead to resistance. These findings highlight the effect of formal education and professional training on AMR awareness.

A review of the literature discloses that most studies in Sri Lanka have focused on healthcare professionals and students, leaving a gap in understanding public awareness and behaviour. By focusing on the general population, this study addresses that gap and contributes new insights into how antibiotic knowledge and practices are evident outside professional settings.

The trend across these studies, alongside our own findings, reveals that AMR awareness differs significantly by population and educational background. In general, individuals with higher formal education, chiefly in healthcare, show better understanding and more appropriate antibiotic-related behaviour. Comparisons between healthcare students and the public in this study further established this inequality and highlighted the need for more inclusive public education efforts.

### Predictors of AMR knowledge

The study revealed several contextual and behavioural factors that significantly predicted the public knowledge of AMR. In the case of rural residence, poor knowledge (AOR=0.15, *P*=0.006) was significantly lower and was in line with studies conducted on a national scale in which rural areas had more misconceptions regarding antibiotics and their relation to AMR [19,20]. In the same way, knowledge of respondents with a monthly income of 20,000–75,000 LKR (AOR=11.10, *P*=0.033) was significantly above average, which illustrates the impact of socioeconomic status on health literacy [21]. One of the behavioural factors that shapes the knowledge of AMR, especially in the context of antibiotics, which is prescribed by a health professional, is self-medication, and so health professional guidance, non-adherence to prescribed medication, is linked with non-prescription self-medication and lower AMR knowledge. These behavioural factors are in line with national data indicating self-medication and non-prescription self-medication to be a major gap in understanding the role of antibiotics [20]. These findings suggest that awareness of AMR is influenced by the supporting and obstructing relationships of socio-structured (socio-structured factors – residence, income) and behavioural (adherence and self-medication) factors. Therefore, public health policies should be aimed at promoting the lower and rural income areas, adherence to professional advice and reduction of non-prescription use of antibiotics to increase awareness and support national goals on controlling and containing AMR.

To contextualize our findings further, we reviewed studies from other South Asian and Asian countries that share social, cultural and economic parallels with Sri Lanka. We then extended our comparisons to more developed, higher-income countries such as the UK and Saudi Arabia to recognize how contradictory healthcare systems, regulatory bodies and levels of public health education affect antibiotic use. These regional and global comparisons revealed the vital roles played by factors such as healthcare system regulation, public education, cultural beliefs and access to antibiotics in influencing antibiotic use behaviours. This broader perspective not only situates our findings within a global AMR narrative but also highlights the importance of tailoring interventions and public health strategies to local contexts.

## Conclusion

The study highlights a significant gap in public knowledge and awareness regarding antibiotic use and antimicrobial resistance in the Western Province of Sri Lanka. In spite of the widespread availability and common use of antibiotics, many lack a clear understanding of their proper use and the possible consequences of misuse. This knowledge gap likely contributes to the overuse and thereby misuse of antibiotics, quickening the development and spread of antibiotic resistance. Addressing this matter is vital to conserving the effectiveness of antibiotics and protecting public health.

Moreover, this study discloses a worrying trend of inconsistent adherence to antibiotic guidelines, irrespective of changing levels of healthcare access and awareness. A considerable portion of the population fails to complete prescribed antibiotic courses, habitually ceasing treatment once symptoms improve. Notably, 71.5% of respondents at first reported completing their prescribed antibiotic course; nevertheless, when asked later in the questionnaire, 65.5% admitted to discontinuing antibiotics prematurely. This discrepancy highlights the disconnect between knowledge and actual practice, even among individuals who obtain antibiotics through healthcare providers.

Comparisons with other studies show changing levels of AMR awareness across different populations. The findings show the impact of education and professional background on AMR knowledge, with individuals in healthcare or formal education settings showing better awareness than the general public. This difference highlights the need for tailored educational strategies to address both general and specialized knowledge gaps.

These findings highlight the need to address not only awareness but also the socioeconomic and structural causes of antibiotic misuse. Public health interventions must go beyond information campaigns to contain community-level education, enhanced regulation of antibiotic access and stricter control of informal dispensing practices. Tailored, context-specific policies and national surveillance systems are indispensable to decreasing inappropriate antibiotic use and combating AMR in Sri Lanka.

Lastly, the insights gained from this study offer valuable guidance for both national and international strategies to combat AMR. At the local level, the findings can support the expansion of targeted public health campaigns, strengthen antimicrobial stewardship programmes and inform evidence-based policy in high-density urban areas like the Western Province. Internationally, they contribute to a larger understanding of the behavioural and structural factors influencing antibiotic misuse, informing global AMR mitigation efforts.

## Supplementary material

10.1099/acmi.0.000945.v5 Supplementary Data Sheet 1.

## Abbreviations

AMR: antimicrobial resistance
AORs: adjusted odds ratios
