# Supplementary material for: Significant gaps in practice present despite higher levels of public awareness in antibiotic use and antimicrobial resistance in the western province of Sri Lanka
Source: Access Microbiol. 2026 May 5;8(5):000945.v5. doi: 10.1099/acmi.0.000945.v5 (PMC13143338; doi:10.1099/acmi.0.000945.v5)
Supplement: Supplementary Data Sheet 1. [file acmi-8-00945-s001.pdf]

SELF- ADMINISTERED QUESTIONNAIRE

Serial No: ....   Date: ....

Public Knowledge and Awareness of Antibiotic Use and Antimicrobial Resistance in the Western Province of Sri Lanka.

This questionnaire is about "Public Knowledge and Awareness of Antibiotic Use and Antimicrobial Resistance in the Western Province of Sri Lanka", we would like you to think carefully about each question and to answer it as honestly as you can. Your name and address do not appear anywhere on this questionnaire. The information that you give will not be used in any way that could identify you.

Section A: Socio demographic factors

01. What is your gender?

    a) Male
    b) Female
    c) Other
    d) Prefer not to say

02. How would you categorize your age?

    a) 18-25
    b) 26-35
    c) 36-45
    d) 46-55
    e) 56 and above

03. In which District of the Western Province do you live in?

    a) Colombo
    b) Gampaha
    c) Kalutara

04. What type of residence do you live in?

    a) Urban

b) Rural

05. What is your marital status?

a) Married
b) Unmarried
c) Divorced
d) Widowed
e) Prefer not to say

06. What is your educational level?

a) Less than O/L
b) O/L
c) A/L
d) Bachelor's degree
e) Postgraduate degree
f) Masters level
g) PHD
h) Prefer not to say

07. What is your occupation?

a) Manager
b) Professionals (Doctor, Nurse, Pharmacist, etc.)
c) Technicians and Associate Professionals
d) Engineer/Technical Professional
e) Clerical Support Workers
f) Self-employed/Entrepreneur
g) Service and Sales Workers (Retail, Hospitality, etc.)
h) Skilled Agricultural, Forestry and Fishery Workers
i) Craft and Related Trades Workers
j) Plant and Machine Operators, and Assemblers
k) Elementary Occupations

l) Armed Forces Occupations
m) Unemployed
n) Prefer not to say

08. Approximately, what is your average monthly income?
a) < 20,000
b) 20,000 – 75,000
c) 75,000 – 200,000
d) >200,000
e) Prefer not to say

Section B: Antibiotic Usage Factors

09. Have you ever used antibiotics? (Medicines that fight bacterial infections, e.g., amoxicillin)
a) Yes
b) No
c) I don't know
d) Don't remember

10. True or false: Antibiotics can kill bacteria.
a) Yes
b) No

11. How do you typically obtain antibiotics?
a) Hospital/healthcare by prescription
b) Retail outlet pharmacy
c) From a friend or family member
d) By sharing with others

12. When prescribed antibiotics, do you generally follow the health professional's advice?
    a) Agree
    b) Disagree

13. Were you provided verbal or written information about how often of the antibiotic to take?
    a) Yes
    b) No

14. Were you provided verbal or written information about how much of the antibiotic to take?
    a) Yes
    b) No

15. Do you make it a point to finish the antibiotic course as advised by the health professional?
    a) Agree
    b) Disagree

16. Do you consider the recommended time gap between antibiotic doses?
    a) Agree
    b) Disagree

17. If you start feeling better after a few days, do you stop taking antibiotics?
    a) Agree
    b) Disagree

18. Have you ever stopped taking antibiotics due to a deviation from the normal schedule?
    a) Agree
    b) Disagree

19. Have you ever stopped taking antibiotics due to negligence, being fed up, or bored?
    a) Agree
    b) Disagree

20. Have you ever taken antibiotics without a prescription?
    a) Yes
    b) No

21. Have you experienced different microbial infections during your lifetime?
    a) Yes
    b) No

22. Have you never suffered from microbial infections?
    a) Yes
    b) No

23. If you've had microbial infections, did you take antibiotics to treat them?
    a) Yes
    b) No

Section C: Antimicrobial Resistance Factors

24. Have you heard or encountered the term “Antimicrobial resistance” before? (When bacteria or other microbes develop the ability to resist the effects of drugs designed to kill them)
    a) Healthcare professional
    b) Mass media
    c) Friends

25. Do you know what the term “Antimicrobial resistance” means?
    a) Yes
    b) No

26. Do you believe that the development of antimicrobial resistance is a problem?
    a) Agree
    b) Disagree

27. Can unnecessary use of antibiotics increase the resistance of bacteria to them?
    a) Agree
    b) Disagree

28. Do you agree that resistance to antibiotics is a worldwide problem?
    a) Agree
    b) Disagree

29. What do you think are the risk factors of antibiotic resistance?

a) Over or underuse of antibiotics
b) Failure to complete the course of therapy
c) Sharing antibiotics with others
d) Taking antibiotics without prescription
e) Taking antibiotics without considering the dose and time gap
f) Others

30. What consequences do you associate with antimicrobial resistance?

a) Decrease antibiotic activity
b) Need for expensive drugs
c) Not cured from diseases
d) Increases intensity and duration of diseases
e) Others
